# Oral and Periodontal Health Status, Peripheral Immune Dysregulation, and Cognitive Impairment in Alzheimer’s Disease: A Clinical and Immunological Study

**DOI:** 10.3390/ijms262311752

**Published:** 2025-12-04

**Authors:** Michał Ochnik, Jacek Zborowski, Jerzy Leszek, Adrianna Senczyszyn, Breno Satler Diniz, Aleksandra Sender-Janeczek, Egbert Piasecki, Marta Sochocka

**Affiliations:** 1 Laboratory of Virology, Department of Immunology of Infectious Diseases, Hirszfeld Institute of Immunology and Experimental Therapy, Polish Academy of Sciences, 53-114 Wroclaw, Poland; m.j.ochnik@gmail.com (M.O.); egbert.piasecki@hirszfeld.pl (E.P.); 2 Department of Periodontology, Wroclaw Medical University, 50-425 Wroclaw, Poland; jacek.zborowski@umw.edu.pl (J.Z.); olasender@poczta.onet.pl (A.S.-J.); 3 Department of Psychiatry, Wroclaw Medical University, 50-367 Wroclaw, Poland; jerzy.leszek@umw.edu.pl (J.L.); adrianna.senczyszyn@umw.edu.pl (A.S.); 4 UConn Center on Aging and Department of Psychiatry, University of Connecticut Health Center, Farmington, CT 06032, USA; diniz@uchc.edu

**Keywords:** periodontal disease, Alzheimer’s disease, inflammation, peripheral immune cells, dysregulation of immune response

## Abstract

Periodontal disease (PeD), a chronic oral infectious-inflammatory condition, has been linked to systemic inflammatory processes, which may contribute to the onset or progression of various systemic disorders including Alzheimer’s disease (AD). We hypothesized that worsening oral and periodontal health, leading to the development of PeD, is associated with cognitive impairment and AD progression as well as peripheral immune system dysregulation. This study included 68 participants: 36 with AD and 32 cognitively healthy, age-matched controls (HCs). Periodontal assessment was performed for diagnosis of PeD (gingivitis or periodontitis). Correlations between oral and periodontal health status, cognitive impairment, and AD severity were evaluated. Peripheral immunity markers were investigated. Peripheral blood leukocytes (PBLs) were stimulated ex vivo with LPS from *Porphyromonas gingivalis* (LPS-PG) to assess cytokine IFN-γ, TNF-α, IL-1β, IL-6, IL-10, and IL-15 production. The average levels of peripheral immunity markers were significantly lower in AD compared to HCs. AD severity was associated with poorer oral hygiene and increased periodontal tissue inflammation. PBLs from AD patients exhibited a baseline impairment in immune responsiveness reflected in decreased spontaneous TNF-α, IL-1β, IL-6, and IL-10 production. Nevertheless, stronger activation in response to LPS-PG was observed. Poorer oral health status in AD was associated with reduced levels of IL-10 and IL-6. Poor oral and periodontal health may contribute to cognitive impairment and AD progression. Even mild inflammation in periodontal tissue or gingivitis may already influence peripheral immune cell conditions, which in turn might be related to negative consequences for the brain and mental health.

## 1. Introduction

Alzheimer’s disease (AD) is the most prevalent form of dementia among older adults. Many hypotheses have been put forward to explain the pathogenesis of AD, but a single, unified theory remains out of reach, likely due to the intricate nature of the disease. Its main pathological characteristics are the buildup of amyloid-β (Aβ) plaques and phosphorylated Tau proteins in neurofibrillary tangles [1]. However, other factors such as immune system dysfunction, neuroinflammation, and intercellular communication abnormalities may also play a role in its development and progression [2]. Recent findings highlight the crucial role of an impaired immune response in both the brain and peripheral systems, with cytokines significantly influencing neurodegenerative processes [3]. However, the full extent of the peripheral immune response in AD is not yet completely understood. It is suggested that disease progression might be influenced by the presence and varying intensity of peripheral inflammation (e.g., due to infection) [4]. Both experimental and epidemiological data support the involvement of infections and microbiome imbalances in the onset of AD [5]. Studies suggest that individuals with a strong systemic inflammatory response are at a higher risk of developing cognitive impairment in the following years, indicating that both bacterial infections and the resulting inflammation may contribute to cognitive impairment [6].

It was postulated that there is an association between periodontal disease (PeD) (especially periodontitis) and AD [7]. Periodontal disease, among the most widespread oral health issues globally, is a chronic infectious-inflammatory condition that targets the tissues supporting the teeth, such as the gingiva and alveolar bone. It encompasses a spectrum from gingivitis—marked by reversible gum inflammation without damage to the periodontal attachment apparatus (cementum, periodontal ligament, and bone)—to periodontitis, a more advanced stage [8]. Gingivitis is highly prevalent, affecting up to 90% of individuals. If left untreated, it may advance to periodontitis, an irreversible condition marked by the destruction of connective tissue, loss of alveolar bone, and ultimately, tooth loss [9,10]. Periodontitis has been linked to systemic inflammatory processes and immune system activation, which may contribute to the onset or progression of various systemic disorders, e.g., chronic obstructive pulmonary disease, pneumonia, rheumatoid arthritis, chronic kidney disease, and AD. The underlying mechanisms potentially include microbial translocation beyond the oral cavity, elevated cytokine production, heightened immune responsiveness, and systemic inflammatory responses, all of which can adversely influence overall systemic health [8]. Moderate to severe periodontitis has been identified as a notable risk factor for the development of dementia. It was suggested that decreased overall oral health and deterioration of periodontal tissues can impact quality of life and worsen patients’ cognitive status [11]. Nevertheless, current research on this association and precise mechanisms linking AD and PeD is still limited and requires further investigation. Importantly, while recent studies provide some lines of evidence that PeD may be related to preclinical AD [12], it still requires verification in independent samples, especially in studies in human populations. Gaining insight into the relationship between PeD and systemic conditions may support the development of comprehensive healthcare approaches that highlight the importance of oral health in enhancing overall systemic outcomes. Additional studies are necessary to better define causal links and evaluate potential therapeutic advantages.

Our study is a comprehensive analysis of the possible association of oral health status, periodontal inflammatory state, and AD. We assessed if the presence of bleeding, which suggests development of inflammation and potential periodontal disease, and its severity are associated with cognitive impairment and AD severity. We also evaluated if the deterioration of periodontal health status, measured with several periodontal disease indexes, may be associated with peripheral immune cell dysfunction ex vivo in AD patients.

## 2. Results

### 2.1. Characteristics of the Study Group

The study involved 68 participants, including the following: 36 individuals (52.9%) with clinically diagnosed Alzheimer’s disease (AD)—patients under the care of the Department of Psychiatry of the Medical University of Wroclaw (MUW). The AD group was aged 44–90 years, comprising 20 women (55.6%) aged 47–82 years and 16 men (44.4%) aged 44–90 years. The control group consisted of 32 cognitively healthy controls (HCs) (47.1%). The HC group was aged 50–80 years, including 18 women (56.3%) aged 51–80 years and 14 men (43.8%) aged 50–75 years. No significant differences were found in the gender distribution between men and women within the AD and HC groups.

Oral hygiene and periodontal health status was assessed in all participants using specific periodontal indexes, such as quantitative evaluation of bleeding on probing (BOP), probing depth of periodontal pockets (PD), clinical attachment loss (CAL), and the amount of dental plaque in interproximal spaces (API, approximal plaque index). Differences in dental hygiene habits were also described using the DMFT measure (Decayed, Missing, and Filled Teeth), which is the sum of teeth with cavities, missing teeth, and those containing fillings. DMFT is a commonly used measure in epidemiology to assess the prevalence of dental caries and evaluate the overall state of oral health [13]. Within the AD group 21 patients had PeD (58%): 15 individuals with gingivitis (41.5%), 6 were diagnosed with periodontitis (17%)—from mild to severe stages—and 15 had a healthy periodontium (41.5%). In contrast, no cases of periodontitis were observed among the cognitively healthy participants (HCs): 9 individuals were diagnosed with gingivitis (28%), while 23 had a healthy periodontal status (72%). Descriptive statistics characterizing age, gender, MMSE, MoCA, AD stage, as well as oral and periodontal health status indexes, and PeD diagnosis for both groups, AD and HC, are presented in Table 1.

Additionally, the study group was characterized regarding dental hygiene habits. Cognitively healthy individuals took better care of their oral health, brushing their teeth twice a day (93.8% of participants), flossing (37.5%), and rinsing (34.4%) their teeth, whereas in the AD group, only 47.2% of participants brushed their teeth twice a day, and the majority did not floss (91.7%) or rinse (94.4%) their teeth. Healthy individuals had, on average, a higher number of fillings (nine teeth) compared to those with AD (six teeth). Both groups had a similar number of teeth, with the HC group averaging 23 teeth and the AD group averaging 22 teeth. In the AD group, 44% of participants had at least one decayed tooth, while in the HC group, 41% of individuals had at least one decayed tooth.

### 2.2. Peripheral Immune Cell Profile and Peripheral Immunity Markers

**Figure 1:**
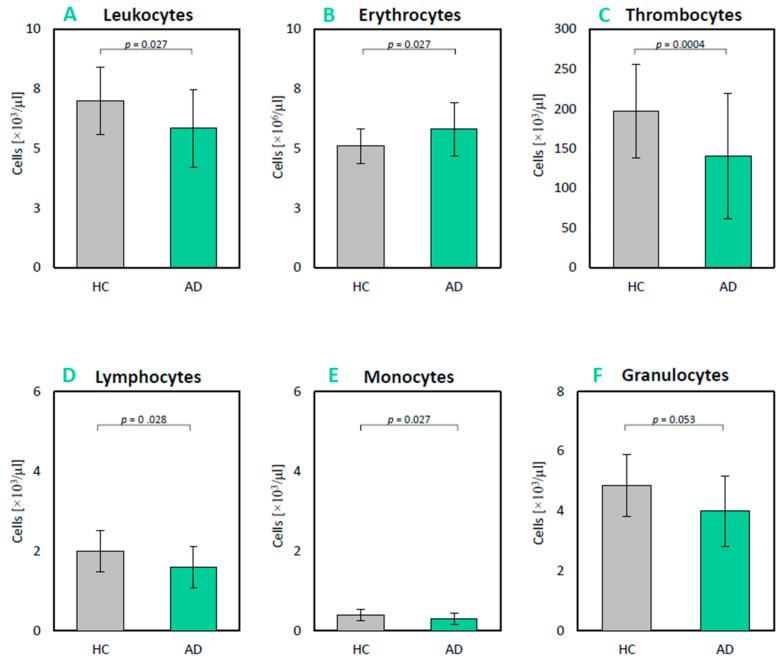
Average values of blood morphology of patients in the study and control groups. Six panels present the average (median) values of blood morphology with the median absolute deviation (MAD): (**A**) leukocytes; (**B**) erythrocytes; (**C**) thrombocytes; (**D**) lymphocytes; (**E**) monocytes; (**F**) granulocytes for the two groups, AD group (n = 36) and HC—control group (n = 32). Statistical significance of differences was tested using the Mann–Whitney U test with *p*-value correction using the Benjamin–Hochberg method. The whiskers represent the SD.

The composition of peripheral innate and adaptive immune cells was analyzed. Basic descriptive statistics for both groups, AD and healthy age-matched controls (HCs), are presented in Table 2 and Figure 1. It was observed that the total number of leukocytes as well as leukocyte subpopulations were reduced in individuals with AD. The average number of white blood cells (WBCs, leukocytes) in the AD group was significantly lower (U = 376.5; *p* = 0.014; *p_(BH)_* = 0.027; r_G_ = −0.30) compared to HC. Furthermore, the number of lymphocytes (U = 391; *p* = 0.023; *p_(BH)_* = 0.028; r_G_ = −0.28) and monocytes (U = 384.5; *p* = 0.016; *p_(BH)_* = 0.027; r_G_ = −0.28) in the AD group was also significantly lower compared to HC. Regarding the number of granulocytes, in AD it was much lower than in HCs, and the result was very close to statistical significance (U = 418; *p* = 0.053; *p_(BH)_* = 0.053; r_G_ = −0.23). In addition, the platelet count was also significantly lower in AD compared to HCs (U = 296.5; *p* < 0.001; *p_(BH)_* = 0.004; r_G_ = −0.41). Non-immune cells—erythrocytes—were significantly increased in AD (U = 768.5; *p* = 0.018; *p_(BH_*_)_ = 0.027; r_G_ = 0.28).

The mean percentage content of individual immune cell populations—lymphocytes, monocytes, and granulocytes—in the AD group was 28.3% (Mdn = 27.6%; MAD = 5.9 percentage points), 4.8% (Mdn = 4.7%; MAD = 0.9 p.p.), and 66.9% (Mdn = 67.7%; MAD = 7.0 p.p.), respectively. In comparison, the corresponding values for the HC group were 29.0% (Mdn = 29.9%; MAD = 3.75 p.p.), 5.8% (Mdn = 5.6%; MAD = 1.3 p.p.), and 65.3% (Mdn = 65.0%; MAD = 3.75 p.p.). A statistically significant difference in percentage content was observed only for monocytes (Student’s *t*-test: *p* = 0.03), whereas no significant differences were found for lymphocytes (Student’s *t*-test: *p* = 0.7) or granulocytes (Student’s *t*-test: *p* = 0.4). The AD group exhibited a lower mean percentage of lymphocytes and monocytes but a higher proportion of granulocytes compared to HC. Additionally, the interquartile range (IQR) was generally greater in the AD group, indicating greater variability in the measured characteristics among patients with AD than among the HCs.

The analysis of peripheral immunity markers revealed differences between patients with AD and healthy controls (HCs). The baseline results are presented in Table 3. There were no statistically significant differences in the GLR (granulocyte-to-lymphocyte ratio) or PLR (platelet-to-lymphocyte ratio) between groups. However, the SII (platelet × GLR) was significantly lower in the AD group compared to HC (*p* = 0.014).

### 2.3. Relationship Between AD Severity, Oral Health, and Periodontal Inflammation

**Figure 2:**
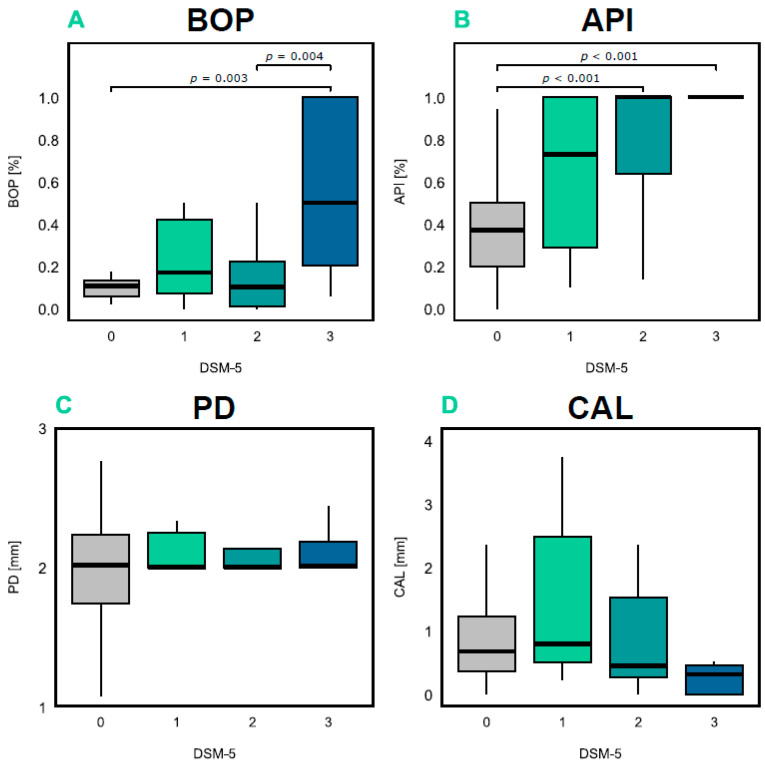
Distribution of clinical periodontal measurements in AD patients depending on the disease severity. The distribution of oral and periodontal indexes is presented in four box plots, where (**A**) BOP variable; (**B**) API variable; (**C**) PD variable; and (**D**) CAL variable. The y-axis represents the values of the indices in their corresponding units, while the x-axis shows the severity of AD according to the DSM-5 classification, where 1—mild AD (n = 7); 2—moderate AD (n = 18); and 3—advanced AD (n = 11). The cognitively healthy controls (HCs) are coded as 0 (n = 32). The p-value for the statistical test is indicated on the plots, with a Benjamin–Hochberg correction applied to the post hoc Dunn test following the Kruskal–Wallis test for four independent groups. Basic descriptive statistics, including the median, upper quartile (Q3), and lower quartile (Q1), are shown in the box. The upper whisker represents the maximum value, and the lower whisker represents the minimum value.

AD is characterized by varying degrees of severity, so the first step was to investigate whether patients with AD differ from each other and cognitively healthy individuals regarding hygiene index (API), inflammation index (BOP), and parameters defining the degree of periodontal tissue destruction (PD, CAL) used in the diagnosis of PeD. The severity of AD was categorized from 1 to 3 using the DSM-5 classification, where 1—mild AD; 2—moderate AD; and 3—advanced AD. Significant differences were found in the distributions of BOP (H(3) = 13.77; *p* = 0.003; η^2^ = 0.17) and API (H(3) = 30.47; *p* < 0.001; η^2^ = 0.43) values. No significant differences were observed for the other variables, PD (H(3) = 3.37; *p* = 0.338; η^2^ = 0.005) and CAL (H(3) = 4.18; *p* = 0.243; η^2^ = 0.02) (Figure 2).

Since patients with AD differ significantly from cognitively healthy individuals in terms of oral hygiene (API) and gingival inflammation (BOP), we decided to investigate whether the progressive deterioration in cognitive function in AD, as measured by the MMSE, correlates with an increased inflammatory burden in periodontal tissues, assessed using the BOP index. BOP is a key clinical indicator of periodontal inflammation and active disease. Bleeding upon probing reflects damage to blood vessels in the gingival tissues, which is a sign of inflammation and may indicate the presence or progression of PeD.

**Figure 3:**
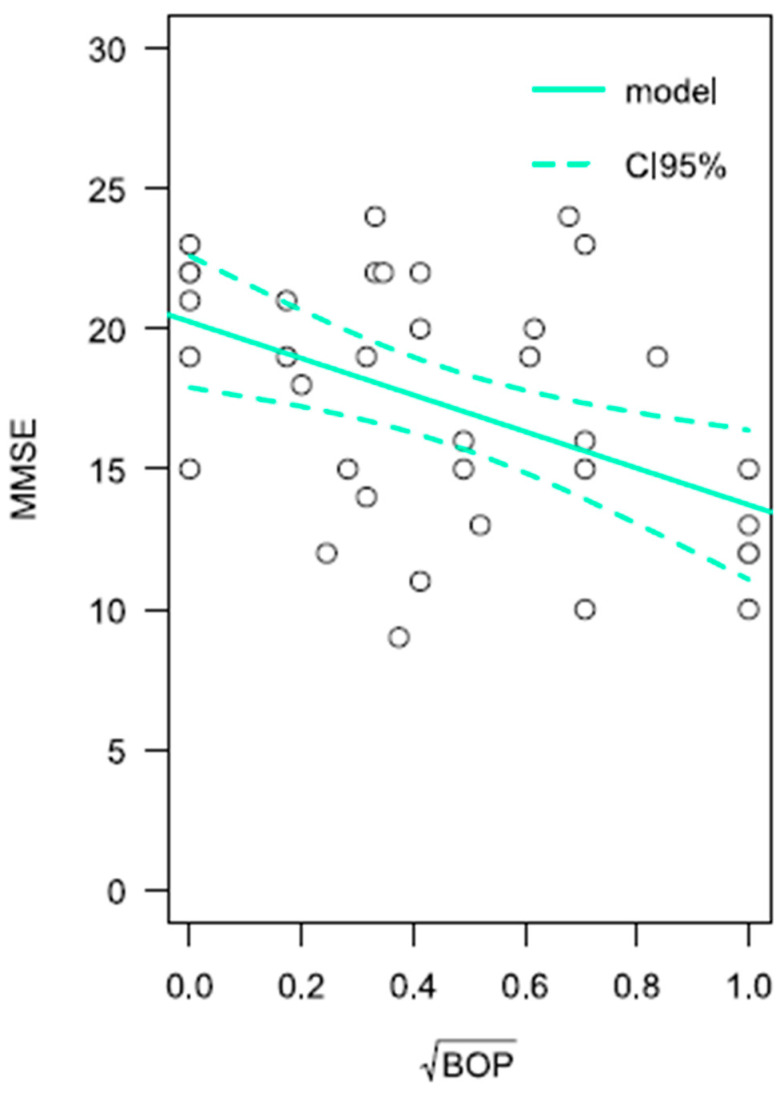
Linear regression analysis of the dependent variable MMSE. On the abscissa axis is the variable BOP on an exponentiation scale; on the ordinate axis is MMSE. The regression model is drawn with a continuous line, while the confidence intervals of the model curve are marked with a dashed line.

The $\sqrt{\mathrm{BOP}}$ index (normalized BOP index transformed into square root form) was significantly associated with MMSE (β = −6522, *p* = 0.004; CI95% = −10,754; −2290) (Figure 3). In the linear regression model for the MMSE variable, in addition to BOP, patient age and sex were also included. Neither sex (*p* = 0.509) nor age (*p* = 0.058) was found to have a significant association with the expected MMSE value.

### 2.4. Activation and Intensity of the Peripheral Immune Cell Response to LPS-PG

Freshly isolated peripheral blood leukocytes (PBLs) ex vivo were stimulated with lipopolysaccharide (LPS) derived from *Porphyromonas gingivalis* (LPS-PG) at a final concentration of 1 μg/mL to evaluate activation of the immune system and its intensity, which can reflect by-production of different cytokines in inflammation. Following 24 h of incubation, the concentrations of selected pro-inflammatory and anti-inflammatory cytokines, including IFN-γ, TNF-α, IL-1β, IL-6, IL-10, and IL-15, were quantified in the supernatants collected from the PBL cultures in response to LPS-PG.

At baseline, significant differences between AD patients and healthy controls were noted in the spontaneous release of cytokines by freshly isolated PBLs, which were not stimulated with LPS-PG. After 24 h of incubation, unstimulated PBLs from AD patients released significantly lower levels of TNF-α (*p_(BH)_* = 0.026), IL-1β (*p_(BH)_* = 0.012), and IL-6 (*p_(BH)_*_=_ 0.012) compared to PBLs from healthy controls (Table 4). Based on Cohen’s index (*d*) scale—an effect size index—it can be seen that the examined differences between groups were greater than “medium”, close to “large”. Importantly, although not statistically significant, it was observed that unstimulated PBLs from AD patients also produced much less IL-10 compared to PBLs from HCs. Following stimulation with LPS-PG, there was an increase in the production of TNF-α, IL-1β, IL-6, and IL-10 but not IFN-γ and IL-15 by PBLs in both AD and control groups, with the final concentrations reaching comparable mean levels in both groups (Table 4). The detailed distribution of cytokine concentration values (pg/mL) for the AD group and the control group (HC) is presented in Table S1 (Supplementary Materials).

Based on the aforementioned observations, the differences in the activation of the immune system and its intensity between HCs and those with AD were evaluated by the introduction of the effect size measure (ES). This represents the natural logarithm of the ratio of cytokine concentrations produced by LPS-PG-treated- to non-treated PBLs, as described by the following formula:

$${\mathrm{ES}}_{j}^{i}\;=\;\ln\left(\frac{\mathrm{treated}}{\mathrm{non}\text{-}\mathrm{treated}}\right)=\ln\left(\mathrm{treated}\right)-\;\ln(\mathrm{non}\text{-}\mathrm{treated}),$$

**Figure 4:**
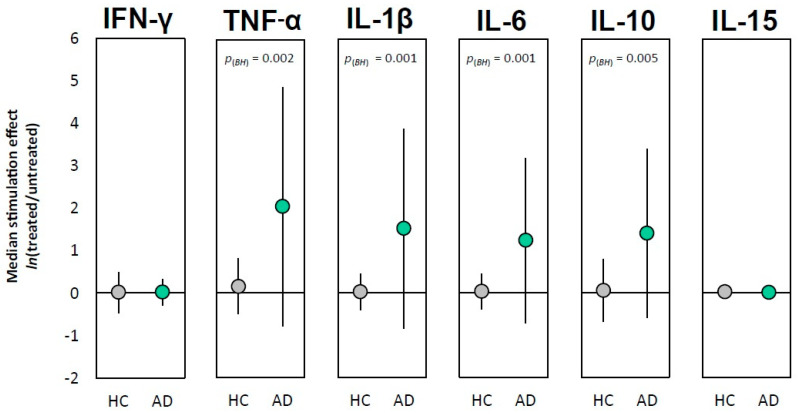
Graphical interpretation of the measured effect size. The points represent the median of the stimulation effect on a logarithmic scale, and the whiskers represent the median absolute deviation (MAD). *p_(BH)_* denotes the *p*-value corrected using the Benjamin—Hochberg method.

Based on the ES measure, it was observed that the activation of PBLs ex vivo was significantly higher in the AD group, which exhibited a greater average increase in cytokine release—TNF-α (U = 311; *p* = 0.001; *p_(BH)_* = 0.002; rG = −0.39), IL-1β (U = 289; *p* < 0.001; *p_(BH)_* = 0.001; rG = −0.43), IL-6 (U = 296; *p* < 0.001; *p_(BH)_* = 0.001; rG = −0.42), and IL-10 (U = 340; *p* = 0.003; *p_(BH)_* = 0.005; rG = −0.35)—compared to the control group. In contrast, the levels of IFN-γ and IL-15 produced after LPS-PG treatment did not differ between the two groups. Results are presented in Figure 4.

### 2.5. Relationship Between AD Comorbidity, Oral Health Status, Periodontal Inflammation, and Peripheral Immune Cell Response

As demonstrated above, PBLs of AD patients exhibited significantly higher cytokine release upon stimulation with LPS-PG. The correlation between AD, oral health status, periodontal inflammation, and the activation of the immune system reflected as the production of cytokines IFN-γ, TNF-α, IL-1β, IL-6, IL-10, and IL-15 in response to LPS-PG was further investigated using multiple linear regression analysis.

**Figure 5:**
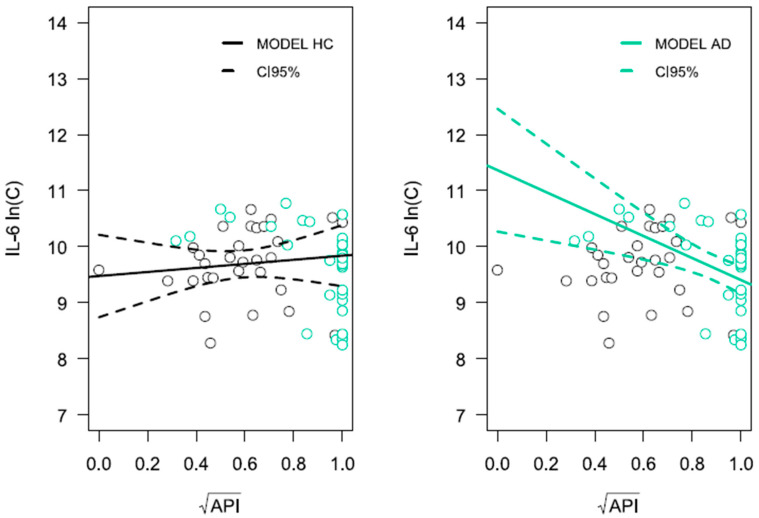
Linear regression analysis for ln(C_IL-10_). The x-axis represents the API variable on a logarithmic scale, and the y-axis represents the natural logarithm of IL-10 concentration. Turquoise circles correspond to observations from the AD group, while the cognitively healthy group is shown in black. The solid line represents the regression model, and the dashed lines represent the confidence intervals for the model curve. Results for LPS-PG-stimulated PBLs are presented.

**Figure 6:**
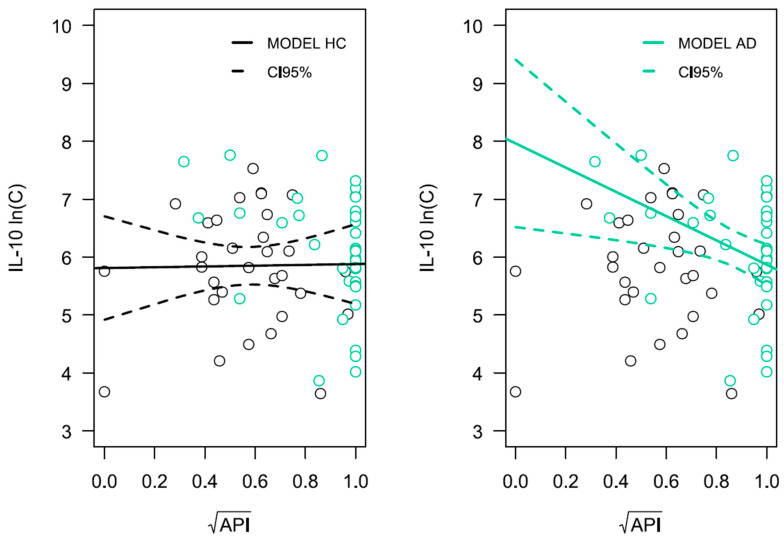
Linear regression analysis for ln(C_IL-6_). The x-axis represents the API variable on a logarithmic scale, and the y-axis represents the natural logarithm of IL-6 concentration. Turquoise circles correspond to observations from the AD group, while the cognitively healthy group is shown in black. The solid line represents the regression model, and the dashed lines represent the confidence intervals for the model curve. Results for LPS-PG-stimulated PBLs are presented.

Significant relationships were observed for the comorbidity of AD, poor oral hygiene, and anti-inflammatory cytokine IL-10 level (Figure 5) as well as pro-inflammatory cytokine IL-6 level (Figure 6), both of which play critical roles in antimicrobial immune response. The level of cytokine IL-10 was influenced by the presence of AD (*p* = 0.024), age (*p* = 0.002), as well as the approximal plaque index (API) in interaction with the presence of AD, denoted as $\sqrt{\mathrm{API}}$ × Alzheimer (*p* = 0.047). It was therefore shown that a higher API value, indicating poorer oral hygiene in AD patients, was associated with lower IL-10 production in response to LPS-PG. Such a relationship was not observed for HCs (*p* = 0.925). The IL-6 concentration was influenced by the presence of AD (*p* = 0.008) and the interaction of variables $\sqrt{\mathrm{API}}$ × Alzheimer (*p* = 0.007). In AD patients, poorer oral hygiene was associated with the production of lower amounts of IL-6 by PBLs following stimulation with LPS-PG cells. Detailed results of linear regression analysis for both ln(C_IL-10_) and ln(C_IL-6_) variables are presented in Table S2 and Table S3, respectively (Supplementary Materials).

## 3. Discussion

Poor oral health and periodontal disease (PeD) extend beyond a localized oral problem—they may significantly impact overall health. The detrimental effects of PeD—an oral chronic inflammatory disease-have been linked to a range of systemic diseases [8]. For over a decade there has been a growing interest in the link between cognitive impairment, dementia, oral health, and PeD. Early studies suggested that inflammation induced by periopathogens may be one of the factors accelerating the development of Alzheimer’s disease (AD) [14], but until now, the relationship between PeD and AD had not yet been fully explored. Investigations into the role of PeD in AD pathology are fully justified. First, there is a strong association of PeD with many systemic diseases [15]. Second, due to the close anatomical location of the oral cavity and the brain, there is the possibility of easy access of oral pathogens and their products to the brain. Finally, the incidence of PeD among AD patients is higher than in the general elderly population [16,17].

In the first step, to evaluate the potential correlation between the deterioration of oral and periodontal health and cognitive impairment, the linear regression analysis was conducted using direct results from a neurocognitive MMSE test and quantitative values of periodontal measurements. Our findings revealed that as cognitive functions deteriorated, the inflammatory burden of periodontal tissues also increased (as indicated by the BOP index). Moreover, we demonstrated that as AD progresses in patients, the value of API increases, indicating worsening oral hygiene status. The lack of correlation between PD and CAL may be due to the low stage of PeD in the study participants or the fact that AD severity is associated with poorer hygiene and increased inflammation rather than more advanced periodontal damage. Nevertheless, this finding suggests that AD severity and inflammation of periodontal tissues are correlated. On the one hand, both PeD and AD are strongly associated with increased inflammatory burden, with AD primarily associated with neuroinflammation but also systemic inflammation, marked by elevated inflammatory markers in the blood, such as IL-1β, TNF-α, and IL-12 [18]. On the other hand, PeD, as an additional source of pro-inflammatory molecules, likely amplifies the systemic inflammatory response, potentially accelerating the progression of AD. Thus, our findings are in line with prior studies pointing out the link between PeD and dementia [19,20,21].

Next, we analyzed the status of the peripheral immune system and activation of peripheral immune cells in response to LPS from *P. gingivalis* (LPS-PG), the main infectious periodontological load. To the best of our knowledge, no studies have explored this mechanism. The selected experimental model of freshly isolated peripheral blood leukocytes (PBLs) ex vivo and the assessment of the inflammatory mediators may provide a more reliable representation of the in vivo immune system condition, immune cell activation, and its intensity. We found that PBLs isolated from AD patients exhibited lower baseline peripheral immune cell activity (unstimulated), as evidenced by the significantly reduced release of pro-inflammatory cytokines TNF-α, IL-1β, and IL-6, but not IFN-γ and IL-15, compared to cognitively healthy controls (HCs). A lower level of IL-10 was also noticed. Exposure of these cells to LPS-PG-initiated immune cell activation and increased secretion of the aforementioned cytokine in both HC and AD patients, ultimately achieving similar concentrations in both groups. However, the intensity of the immune response was significantly stronger in AD patients compared to the HCs, as demonstrated by the effect size (ES) measure calculated for both groups. In this study, a lack of LPS-PG influence on IL-15 production by PBLs was surprising, as many inflammation stimuli increase blood levels of IL-15, including LPS [22]. However, in peripheral macrophages and monocytes, IL-15 is also induced by IFN-γ, and in our study no effect of LPS-PG was observed on IFN-γ production. Interestingly, in a previous study using a viral stimulant, we observed an increase in the production of the cytokines TNF-α, IL-1-β, and IL-10 in both AD and HC groups, but that increase (the effect size measure, ES) was significantly greater in HC [23], in contrast to the study presented here using LPS-PG. Moreover, an increase in IFN-γ production by PBLs from the HC group in response to viral infection was also indicated. However, we did not observe an IFN-γ response from PBLs of AD patients. In addition, our previous research indicated that, as well as with IFN-γ, PBLs from AD patients did not release higher amounts of IFN-α in response to viral infection compared to HCs. PBLs from AD patients were unable to generate a strong antiviral cytokine response [23]. Further analyses are therefore needed to determine whether the lack of IFN-γ production in response to both viral and bacterial antigens indicates a disruption in the interferon signaling pathway. It therefore appears that PBLs from AD patients respond specifically to a given stimulus, but a common observation across both studies is the lower baseline levels of cytokines released by unstimulated cells.

Although the increase in cytokine production following LPS-PG stimulation is an expected outcome (regardless of the origin of immune cells), in our study the most critical finding is the effect size, which highlights significant differences between AD and HCs, indicating an altered immune response pattern in patients with AD. The observed effects may be, on one hand, explained by the immunological exhaustion of T cells, a dysfunctional state in which chronically activated T cells lose their normal abilities. In our studies, cells isolated from AD patients exhibited lower spontaneous production of inflammatory molecules compared to leukocytes of HCs. It was shown recently by Grayson et al. [24] that amyloid-positive mild cognitive impairment (APMCI) participants exhibited a significant increase in T cells that did not produce cytokines after restimulation and showed elevated levels of PD-1 and Tox, indicating that these cells are likely exhausted. On the other hand, we noticed that PBLs from AD participants responded more robustly to LPS-PG, suggesting signs of hyperactivation (hyperresponsiveness) that may lead to excessive inflammation. The lower baseline functionality of peripheral immune cells and changed pattern of immune response observed in AD patients may also be explained by the characteristic peripheral immune cell profile and peripheral immunity markers such as GLR (granulocyte-to-lymphocyte ratio), PLR (platelet-to-lymphocyte ratio), and SII (systemic immune-inflammation index), reflecting the balance between innate and adaptive immunity as proposed by Willik et al. [25]. They indicated the role of an imbalance in the immune system towards innate immunity in the pathogenesis of dementia. In our study, we clearly demonstrated significantly reduced values of total white blood cells (WBCs)—leukocytes—as well as their individual subpopulations, such as granulocytes, lymphocytes, and monocytes, in patients with AD. Additionally, a markedly lower platelet count was observed in AD. Considering the examined immunological indices, we found a decrease in PLR values and a significant reduction in SII (measured as platelet × GLR) in AD patients. The same hematological cellular profile (decreased peripheral immune cell count) and inflammation indices in patients with AD have been demonstrated [26,27], although these studies primarily focused on the correlation of these ratios with disease severity and not with peripheral immune system functionality. Curiously, other studies indicated that the SII in patients with AD was higher than in healthy controls [27,28,29], contrasting with our findings; although in the current study, SII was measured based on granulocyte counts. However, considering the peripheral immune cell dysfunction observed in our study, a lower SII appears to be a logical outcome. A high SII is a marker of significant inflammation, which is often a poor prognosis in various diseases including cancer and cardiovascular issues [30,31]. We assume that our finding of a lower SII is logical within the framework of observed immune exhaustion/suppression in AD patients, suggesting an impaired, but at the same time very reactive in the context of bacterial or viral pathogens, systemic immune response, as opposed to chronic active inflammation. Moreover, peripheral immune dysfunction that lowers the overall systemic inflammatory signal or suppresses cell counts would naturally correlate with a lower SII score. This highlights that perhaps immune changes in AD can be non-linear and may depend on the disease stage.

In our research on the activation and response of PBLs, the choice of bacterial antigen (LPS-PG) was also important, as we focused on determining the role of oral and periodontal health status in AD. In primary studies to analyze the functionality of PBLs in AD and the role of periodontal inflammation, we selected the classical bacterial antigen derived from *Escherichia coli* (LPS-EC) [32]. We demonstrated that LPS-EC stimulation resulted in a significant increase in the production of the cytokines TNF-α, IL-1β, IL-6, and IL-10. Comparing these two studies, we observed that LPS-EC stimulation led to the secretion of generally higher amounts of cytokines than LPS-PG, indicating that using LPS from different bacterial sources is associated with distinct immune cell responses in AD patients.

Our next step in this comprehensive analysis of the interplay between oral and periodontal health status and AD was the assessment of the immune system condition and status of PBLs in AD patients considering periodontal parameters. We showed functional differences in PBLs between HCs and AD. It was revealed that the ability of PBLs from AD patients to produce the anti-inflammatory IL-10 and pro-inflammatory IL-6, which play key roles in the response to bacterial antigens, was closely associated with the value of the API. A higher API value, indicating poorer oral hygiene, was linked to lower concentrations of these cytokines. Such a relationship was not observed in the HCs. This is a very interesting result, as the link between API and PeD development is well-known and dental plaque is considered as a causative factor of gingivitis and periodontal inflammation [33]. Furthermore, certain bacteria, such as *P. gingivalis*, may have the ability to reduce the secretion of anti-inflammatory cytokines, including IL-10 [34]. Authors of this study indicated that PBMCs from patients with PeD exhibited reduced production of anti-inflammatory cytokines. Monocytes, being a key source of IL-10 production, may have a regulatory role in the pathogenesis of periodontal inflammation through the IL-10 they produce. In our study, we found significantly lower monocytes in AD patients compared to HCs. And as shown in the studies of Luo et al., lower monocytes are associated with a higher risk of developing AD [35], which confirms the important role of the peripheral innate immune system in the pathogenesis of the disease.

Our study has several strengths. A key strength is its comprehensive approach, which integrates the analysis of neurocognitive and periodontal clinical indicators, as well as the composition of peripheral immune cells and activation status of the immune system. This interdisciplinary approach enhances our understanding of the potential mechanisms underlying the comorbidity of AD and periodontal inflammation. Additionally, the application of an advanced statistical approach to jointly analyze clinical and immunological parameters represents another significant strength, as it enables us to test the proposed hypotheses effectively. Furthermore, our findings should inform the design of future scientific, experimental, and clinical studies aimed at investigating whether PeD treatment could have a beneficial effect on immune system functioning, mitigate immunological deficits, and improve patients’ quality of life. Ultimately, such interventions could potentially reduce or slow the progression of cognitive disorders and AD.

However, our study also has some limitations. The study group consists solely of individuals from southern Poland (single-center study), which may limit the generalizability of our findings to other populations of AD patients and cognitively healthy elderly individuals worldwide. The relatively small sample size may affect the robustness and clarity of the results, as well as the accuracy of the conclusions drawn from the data. To minimalize the effect of this limitation on final conclusions, Benjamin–Hochberg correction was used in data analysis to control the false discovery rate (FDR). Adjustments were made for individual groups of statistical tests involving the same family of hypotheses. A popular approach was used in which the *p*-value was modified to compare it with a given error rate controlled at the alpha level. In addition, patients were diagnosed for the presence of gingivitis and periodontitis; however, due to the small sample size, the severity of periodontitis and risk categories (grades) were not included in the data analysis. Our focus was primarily on determining whether poor oral health and the progression of periodontal tissue disease, reflected by worsening periodontal indices, are associated with cognitive impairment, advanced AD, and dysfunction of peripheral immune cells. Further studies incorporating the severity of periodontitis are necessary to confirm these findings and to elucidate the potential link between PeD and AD. The cross-sectional nature of the study also makes it impossible to indicate cause–effect relationships between the two diseases. Furthermore, the analyses did not take into account the confounding influence of coexisting somatic diseases, applied pharmacotherapy, or diet. During recruitment, however, the presence of serious inflammatory diseases, including diabetes, metabolic diseases, and autoimmune diseases, as well as recent infections or cancers, was excluded.

In summary, AD is associated with significant changes in the profile of peripheral immune cells, with the overall number and specific leukocyte subpopulations significantly reduced compared to cognitively healthy individuals. We suggest that this may be one of the reasons for the weakened immune response to bacterial and viral antigens and indicate potential immunological deficiencies in this group of patients. In the study aimed to identify a robust immune-related signature of PeD and AD, immune-related biomarker genes DUSP14, F13A1, and SELE were indicated. In addition, immune cells such as macrophages M2 and NKT, B-cells, CD4+ memory T-cells, and CD8+ naive T-cells emerged as key immune cells linking PD with AD [36]. To date, some studies conducted demonstrate a positive association between AD and PeD, while others do not, likely due to differences in PeD severity across the studied populations or differences in study designs [37,38]. Future research directions: Understanding the precise mechanisms associated with impaired innate and adaptive immune responses is a further direction of research in this area. We are currently conducting experimental and clinical studies to demonstrate whether PeD treatment in AD patients can beneficially impact the peripheral immune system, reduce immunological deficits, and improve quality of life. In the future, the results of the research presented here should also be used to raise public awareness of oral health and hygiene as an important preventative factor in the fight against dementias such as AD, in particular given recent findings indicating that PeD is strongly associated with an increased risk of AD [39]. The presence and severity of PeD should be considered when estimating the likeliness of progression to AD in preclinical populations, as well as when developing questionnaires or clinical tools intended to assess this risk.

## 4. Materials and Methods

### 4.1. Ethical Issues

These studies were ethically approved—consent No. KB—76/2019 issued by the Bioethics Committee at the Medical University of Wroclaw on 12 February 2019. The study was performed in accordance with the ethical standards as laid down in the 1964 Declaration of Helsinki and its later amendments or comparable ethical standards. Only those individuals who gave their consent themselves or through their guardians participated in the study.

### 4.2. Study Group

The study group consisted of 68 participants: 36 patients with Alzheimer’s disease (AD) and 32 cognitively healthy individuals (HCs). AD patients were recruited by clinicians from the Department of Psychiatry of the Medical University of Wroclaw (MUW). Cognitively healthy individuals were volunteers who declared that they did not experience any memory problems and also did not show any signs of cognitive decline. They were not under the care of the Department of Psychiatry at MUW or any other memory clinic. Also, they did not report other neurological disorders.

### 4.3. Clinical Examination

Neurocognitive assessment: AD patients (n = 36) underwent detailed neuropsychological tests regarding the inclusion and exclusion criteria from the study. For this purpose, interviews with patients and their caregivers were conducted by clinicians, as well as tests assessing the degree of cognitive impairment, including the MMSE (Mini-Mental State Examination), the MoCA (Montreal Cognitive Assessment) test, and the Sunderland clock drawing test. Inclusion criteria: Adults (≥ 45 years). For cognitive impairment and AD group—the diagnosis of AD (MoCA and MMSE scores ≤ 24 pts). Exclusion criteria: included age over 90 years (for both groups), diagnosed types of dementia other than AD or other concomitant psychiatric diseases, and recent brain infection. Additional criteria for AD and controls (HCs) included addiction or abuse of alcohol and psychoactive substances in the last 2 years, smoking tobacco products, taking antibiotics in the last 6 months or having an infectious disease in the last 3 months, previous stroke or cancer, diabetes, obesity, and autoimmune or chronic inflammatory diseases.

Periodontal assessment was performed on all study participants (n = 68) at the Department of Periodontology (MUW). Physical examination of the oral cavity was performed by one clinician, an experienced specialist periodontist using a dental mirror and a CP-15 periodontal probe (Hu Friedy, Chicago, IL, USA). The number of teeth was measured, excluding third molars, and the Decayed, Missing, and Filled Teeth Index (DMFT) was determined. After staining the teeth with a staining solution, oral hygiene status was determined based on the Lange approximal plaque index (API). Pocket Depth (PD) and clinical attachment loss (CAL) were measured at six disto-, mesio-, and mesiobuccal locations and in lingual positions of all teeth, except third molars, and bleeding on probing (BOP) was measured at four measurement points and expressed as a percentage of bleeding area. Any changes in the oral mucosa were also recorded.

The 2017 World Workshop on the Classification of Periodontal and Peri-Implant Diseases and Conditions was utilized, which reflects the updated framework jointly adopted by the American Academy of Periodontology (AAP) and the European Federation of Periodontology (EFP) [40]. Periodontal health was defined as the absence of clinically detectable inflammation, with probing depths (PD) ≤3 mm, no clinical attachment loss (CAL), and bleeding on probing (BOP) present in <10% of sites, consistent with the criteria proposed by Tonetti et al. [41]. Gingivitis alone was operationally defined as the presence of BOP in ≥10% of sites without radiographic evidence of alveolar bone loss and with clinical attachment levels within normal limits. Periodontitis was diagnosed according to the case definition proposed by the 2017 AAP/EFP classification, which requires interdental CAL detectable at ≥2 non-adjacent teeth, or buccal or oral CAL ≥ 3 mm with pocketing > 3 mm detectable at ≥2 teeth. Furthermore, all three stages of severity—Stage I (mild), Stage II (moderate), and Stage III/IV (severe)—were incorporated in the diagnostic criteria.

During the study, an interview was conducted in which patients were asked about their oral hygiene habits, such as the frequency of brushing and flossing teeth and mouth washing. For further linear regression analysis, assessed periodontal indicators reflecting deterioration in oral and periodontal health status were used.

### 4.4. Hematological Blood Sample Analysis and Peripheral Immunity Markers

Immune cells profiles were determined qualitatively and quantitatively using a Mythic 18 hematology analyzer (Cormay Diagnostic, Warsaw, Poland). For this purpose, 50 µL of blood sample was transferred to a 0.5 mL centrifuge tube, which was then analyzed using the analyzer according to the manufacturer’s instructions. The test was performed within 2 h after the material was collected. Laboratory measurements included absolute counts of white blood cells (leukocytes), monocytes, granulocytes, platelets, and lymphocytes per microliter (10^3^/µL). The neutrophil counts were unavailable, thus the granulocyte count was used as a reliable proxy, as neutrophils are the most abundant type of granulocyte [42,43]. Several peripheral immunity markers, i.e., granulocyte-to-lymphocyte ratio (GLR) and platelet-to-lymphocyte ratio (PLR), were calculated by dividing the granulocyte count by the lymphocyte count and the platelet count by the lymphocyte count, respectively. The systemic immune-inflammation index (SII) was defined as the platelet count multiplied by the GLR.

### 4.5. Isolation of Peripheral Blood Leukocytes (PBLs)

Peripheral blood samples from all participants were taken into heparin tubes. The whole population of white blood cells, PBLs, was isolated within two hours of collection, according to a standard protocol, from 8 mL of peripheral blood by gradient centrifugation in Gradisol G (Aqua-Med, Łódź, Poland) and maintained in RPMI 1640 medium (HIIET, Wroclaw, Poland) with antibiotics (100 U/mL penicillin and 100 μg/mL streptomycin), 2 mM L-glutamine, and 2% FBS (all from Sigma-Aldrich, St.Louis, MO, USA). This method allows the achievement of high purity and yield without erythrocyte contamination.

### 4.6. Lipopolysaccharide from Porphyromonas gingivalis

Lipopolysaccharide from *P. gingivalis* (LPS-PG) was purchased from Invivogen (Toulouse, France). The lyophilized powder was reconstituted in 1 mL of ultra-pure water and then aliquoted. Next it was stored at −20 °C.

### 4.7. PBL Culture and Stimulation with LPS-PG

Freshly isolated PBLs of each participant were divided into two parts. One part (1 × 10^6^ cells mL^−1^) was suspended in PRMI medium with 2% of fetal bovine serum (FBS) and incubated for 24 h at 37 °C/5% CO_2_ (untreated). The second part of the PBLs (1 × 10^6^ cells mL^−1^) was suspended in PRMI medium with 2% FBS and stimulated with 1 μg mL^−1^ of LPS-PG. Next cells were incubated for 24 h at 37 °C/5% CO_2_. Samples of medium above untreated and treated PBLs were collected after 24 h of incubation and stored at −80 °C for cytokine determination.

### 4.8. Quantitative Cytokine Measurement

The concentrations of six cytokines, IFN-γ, TNF-α, IL-1β, IL-6, IL-10, and IL-15, were measured in the supernatants collected from leukocytes (PBLs) that were unstimulated and 24 h-stimulated with LPS-PG. Measurements were performed using the ELISA immunoenzymatic method with ready-to-use kits from BD Biosciences (San Diego, CA, USA) according to the manufacturer’s instructions.

### 4.9. Statistical Analysis

Sample size calculation. Based on Monte Carlo simulation with B = 1000 repetitions, the power of the statistical test at significance level 0.05 was calculated to estimate the number of patients planned to be included into the study. The minimum number of patients that should complete the study was *n* = 65. The estimated power of the test for recruited number of patients was 80% for *n* = 68. We estimated the sample size based on our preliminary results and the expected effect sizes of the differences in cytokine levels between individuals with AD and PeD vs. AD without PeD.

The results were analyzed statistically and visualized using the integrated programming environment R Studio 2024.04.2 build 764 for the R language (version 4.4.1). For all variables significantly deviating from the normal distribution, the Box–Cox transformation was applied. In order to assess significance of the differences between two independent groups, Student’s *t*-test was performed for variables with equal variances, Welch’s t test for data with unequal variances, or in the case of samples for which the distribution of comparable variables did not meet the assumptions of normality, the Mann–Whitney U test was performed. When comparing more than two independent groups, in the case of data that did not meet the assumptions of normal distribution, the Kruskal–Wallis rank test and Dunn’s post hoc test were performed. The influence of PeD on cytokine response was examined using multiple (multivariable) linear regression. Each model was assessed for the presence of influential observations using the Cook distance measure. Model diagnostics included assessment of model linearity tested with the Wald–Wolfowitz series test, normality of the random component tested with the Shapiro–Wilk test, and homoscedasticity of the random component tested with the Harrison–McCabe test. Effect size indicators were used to supplement the inference procedure.

A test probability level of α = 0.05 was assumed. The first type error was controlled using *p*-value corrections using the Benjamin–Hochberg method. The square root transformation of API and BOP values was applied to meet the assumptions of linear regression, specifically the requirement for normally distributed residuals and homoscedasticity. The BOP values exhibited a positively skewed distribution, which could bias the estimation of regression parameters if uncorrected. Square root transformation is a well-established statistical technique for stabilizing variance and reducing skewness, especially for proportion- or count-based biological variables. Importantly, this transformation does not alter the direction or significance of correlations with the dependent variable but instead adjusts the scale of measurement to achieve a better model fit.

## 5. Conclusions

Poor oral hygiene and Alzheimer’s disease (AD) appear to be interconnected in a bidirectional manner. Cognitive impairment often leads to diminished oral care, increasing the risk of developing periodontal disease (PeD). Conversely, emerging evidence indicates that PeD itself may play a role in the onset or progression of AD. However, the relationship between these two conditions remains complex and not fully understood. Our study contributes to the growing evidence that poor oral health and chronic peripheral infections, such as PeD and related inflammation, influence cognitive deterioration and AD severity and place a persistent burden on the aging immune system. Despite the fact that advanced stages of periodontitis were not predominant in the studied AD group, a correlation was still observed, which is not an obvious conclusion. This suggests that even mild inflammation or gingivitis may influence peripheral immune cell conditions. This may, in turn, lead to sustained immune activation and the breakdown of proper immune regulation. When peripheral immune cells become dysfunctional—exhibiting both exhaustion and hyperreactivity in response to infectious antigens—they may negatively influence brain tissue and in turn contribute to the initiation and/or exacerbation of neuroinflammation. It is well-established that during aging the increased permeability of the blood–brain barrier (BBB) allows peripheral immune cells to access the brain, actively participate in immune processes within the central nervous system, and potentially contribute to the development of clinical AD. This highlights the need for further research into the clinical and therapeutic significance of these findings.

## Figures and Tables

**Table 1:** Descriptive statistics characterizing age, gender, and clinical scales to assess dementia, as well as oral health and periodontal status indexes in the study group.

Variable	Group	Mean	SD	Min–Max
Age	AD	68	11	44–90
	Control	63	7.77	50–80
Gender	Group	Women	Men	% Men
	AD	20	16	44.4%
	Control	18	14	43.7%
	Clinical parameters to assess dementia			
MMSE	AD	Mean	SD	Min–Max
	Women	17.6	4.3	10–23
	Men	16.9	4.8	9–24
MoCA	AD	Mean	SD	Min–Max
	Women	14.1	3.7	5–22
	Men	14.1	5.3	4–24
DSM-5	AD	Mild	Moderate	Severe
	Women	3	12	5
	%	15.0%	60.0%	25.0%
	Men	4	6	6
	%	25.0%	37.5%	37.5%
	Clinical parameters to assess periodontal disease			
BOP [%]	Group	Mean	SD	Min–Max
	AD	31	34	0–100
	Control	13	16	2–88
API [%]	Group	Mean	SD	Min–Max
	AD	82	28	10–100
	Control	39	25	0–100
PD [mm]	Group	Mean	SD	Min–Max
	AD	2.2	0.5	1.0–3.6
	Control	2	0.4	1.1–2.8
CAL [mm]	Group	Mean	SD	Min–Max
	AD	1.1	1.4	0.0–5.0
	Control	1.2	1.4	0.0–6.3
DMFT	Group	Mean	SD	Min–Max
	AD	18	7	1–28
	Control	15.8	5.6	3–27
Teeth	Group	Mean	SD	Min–Max
	AD	19	8.6	2–28
	Control	21	5	7–28
PeD	Group	Healthy	Gingivitis	Periodontitis
	AD	15	15	6
	%	41.5	41.5	17
	Control	23	9	0
	%	72	28	0

SD—Standard Deviation; MMSE—Mini Mental State Examination; MoCA—Montreal Cognitive Assessment; DSM-5—Diagnostic and Statistical Manual of Mental Disorders; BOP—Bleeding on Probing; API—Approximal Plaque Index; PD—Pocket Depth; CAL—Clinical Attachment Loss; DMFT—Decayed, Missing, and Filled Teeth, PeD—Periodontal Disease.

**Table 2:** Basic descriptive statistics of peripheral immune cell profile.

Group	Variable		Min	Q1	Mdn	MAD	Q3	Max
AD	leukocytes	×10^3^/µL	3.8	4.8	5.9	1.1	6.8	10.4
	lymphocytes	×10^3^/µL	0.8	1.3	1.6	0.4	2.0	3.1
	monocytes	×10^3^/µL	0.1	0.2	0.3	0.1	0.4	0.5
	granulocytes	×10^3^/µL	2.2	3.2	4.0	0.8	4.7	7.1
	lymphocytes	%	15.3	21.3	27.6	5.9	31.3	52.4
	monocytes	%	2.5	3.8	4.7	0.9	5.5	10.0
	granulocytes	%	44.0	64.0	67.7	7.0	75.2	78.4
	thrombocytes	×10^3^/µL	59.0	85.8	141.0	53.0	183.0	332.0
HC	leukocytes	×10^3^/µL	4.2	5.4	7.0	0.95	7.7	10.9
	lymphocytes	×10^3^/µL	0.9	1.6	2.0	0.35	2.2	3.6
	monocytes	×10^3^/µL	0.0	0.3	0.4	0.10	0.5	0.8
	granulocytes	×10^3^/µL	2.3	3.6	4.9	0.70	5.2	7.0
	lymphocytes	%	13.8	25.7	29.9	3.75	33.1	38.9
	monocytes	%	0.6	4.4	5.6	1.30	7.1	10.3
	granulocytes	%	52.9	61.5	65.0	3.75	68.7	82.6
	thrombocytes	×10^3^/µL	102.0	155.0	197.0	40.00	231.3	314.0

Min—minimum value; Q1 and Q3—lower and upper quartiles; Mdn—median; MAD—absolute deviation from the median; Max.—maximum value.

**Table 3:** Peripheral immunity markers derived from hematological parameters.

Index	Group	Min	Q1	Mdn	MAD	IQR	Q3	Max	*p*
GLR	AD	0.84	2.06	2.51	1.20	1.48	3.53	4.88	0.530
	HC	1.38	1.87	2.21	0.62	0.78	2.65	6.22	
PLR	AD	20.69	57.27	81.22	54.21	71.39	128.67	218.89	0.078
	HC	38.61	81.74	100.75	38.55	52.53	134.28	225.00	
SII	AD	86.71	233.37	299.69	176.87	289.99	523.37	1001.00	0.014
	HC	221.40	343.40	438.60	129.62	176.35	519.70	1079.20	

IQR—interquartile range; MAD—median absolute deviation; Q1—the first quartile; Q3—the third quartile; GLR—granulocyte-to-lymphocyte ratio; PLR—platelet-to-lymphocyte ratio; SII—systemic immune-inflammation index (platelet × GLR).

**Table 4:** Comparison of cytokine production by unstimulated and LPS-PG-stimulated PBLs with Welch’s *t*-test for differences between groups.

Cytokine		HC		AD					95% CI		
		M	SD	M	SD	t	df	*p_(BH)_*	LL	UL	*d*
IFN-γ	unstimulated	2.07	0.97	2.17	0.47	−0.54	43.4	0.594	−0.48	0.28	−0.14
	LPS-PG-stimulated	2.14	0.92	2.21	0.52	−0.37	48.0	0.712	−0.44	0.30	−0.09
TNF-α	unstimulated	5.07	1.60	3.89	2.18	2.55	63.9	0.026	0.26	2.10	0.61
	LPS-PG-stimulated	5.81	0.99	6.14	0.90	−1.45	63.3	0.153	−0.79	0.13	−0.35
IL-1β	unstimulated	5.57	1.42	4.07	2.38	3.21	58.2	0.012	0.57	2.44	0.76
	LPS-PG-stimulated	6.01	0.80	6.17	0.57	−0.94	55.1	0.351	−0.50	0.18	−0.23
IL-6	unstimulated	8.99	1.44	7.24	3.12	3.02	50.5	0.012	0.59	2.91	0.71
	LPS-PG-stimulated	9.57	0.80	9.57	0.79	−0.02	64.9	0.981	−0.39	0.38	−0.01
IL-10	unstimulated	5.28	1.46	4.55	2.13	1.66	62.2	0.153	−0.15	1.60	0.39
	LPS-PG-stimulated	5.85	1.01	6.10	1.00	−1.04	64.9	0.302	−0.74	0.23	−0.25
IL-15	unstimulated	3.39	0.09	3.35	0.22	1.06	47.7	0.353	−0.04	0.12	0.25
	LPS-PG-stimulated	3.40	0.10	3.36	0.22	0.89	48.3	0.376	−0.04	0.11	0.21

The table presents transformed data x’ = ln(x). To obtain the original values, re-transformation x = ex’ should be performed. The significance of differences between groups was tested using Welch’s *t*-test for independent samples with different variances. HC—group of cognitively healthy individuals (n = 32); AD—group of individuals with Alzheimer’s disease (n = 36); n—number of observations; M—arithmetic mean of normalized data (transformation: natural logarithm); SD—standard deviation; t—value of the test statistic; *p*—test probability value for the *t*-test; CI—confidence interval for the difference between the means; LL and UL—lower and upper limits of the confidence interval; *d*—Cohen’s d effect size index.

## Data Availability

The raw data supporting the conclusions of this article will be made available by the authors upon request.

## Supplementary Materials

The supporting information can be downloaded at https://www.mdpi.com/article/10.3390/ijms262311752/s1.
