# Supplementary material for: Oral and Periodontal Health Status, Peripheral Immune Dysregulation, and Cognitive Impairment in Alzheimer’s Disease: A Clinical and Immunological Study
Source: Int J Mol Sci. 2025 Dec 4;26(23):11752. doi: 10.3390/ijms262311752 (PMC12693382; doi:10.3390/ijms262311752)
Supplement: Supplementary file 1 [file ijms-26-11752-s001.zip › ijms-4022049-supplementary.pdf]

Table S1. Distribution of cytokine concentration values for the AD group and HC group

Group	Cytokine		*Min.*	*Q1*	*Mdn.*	*MAD*	*Q3*	*Max.*
			[pg/ml]					
AD	IFN-γ	unstimulated	4.0	6.8	8.5	1.9	10.5	31.1
		LPS-PG stimulated	2.4	7.3	8.7	2.0	11.1	39.9
	TNF-α	unstimulated	2.9	7.1	25.3	20.6	278.8	2241.4
		LPS-PG stimulated	59.4	250.4	544.8	298.9	843.1	2136.5
	IL-1β	unstimulated	2.0	3.8	104.1	101.7	688.0	892.7
		LPS-PG stimulated	119.4	353.6	528.6	247.2	796.7	863.7
	IL-15	unstimulated	20.4	23.9	27.9	4.9	33.0	47.4
		LPS-PG stimulated	20.9	23.8	28.6	4.9	33.3	45.8
	IL-6	unstimulated	0.4	107.9	6072.9	6058.1	18183.4	38169.8
		LPS-PG stimulated	3817.0	8100.4	17430.4	8578.7	24745.7	47767.8
	IL-10	unstimulated	2.2	11.9	105.4	100.6	616.2	2332.8
		LPS-PG stimulated	47.7	258.6	461.6	304.5	864.3	2345.0
HC	IFN-γ	unstimulated	0.4	7.4	9.4	2.8	12.6	32.3
		LPS-PG stimulated	0.6	7.2	10.1	3.2	15.2	24.5
	TNF-α	unstimulated	4.6	61.8	209.9	175.0	505.8	2585.3
		LPS-PG stimulated	23.2	200.8	367.1	216.4	639.8	2295.7
	IL-1β	unstimulated	1.6	167.7	421.0	280.2	740.8	816.0
		LPS-PG stimulated	51.7	252.9	578.0	229.0	772.1	890.1
	IL-15	unstimulated	23.7	28.9	30.4	1.4	31.2	35.4
		LPS-PG stimulated	24.3	28.9	30.3	1.6	31.8	34.3
	IL-6	unstimulated	34.3	4487.1	11756.6	8727.3	20669.9	40660.1
		LPS-PG stimulated	1750.1	11511.8	16479.0	6954.5	25766.1	42785.9
	IL-10	unstimulated	14.9	57.2	231.6	190.1	828.1	1960.2
		LPS-PG stimulated	38.2	210.2	337.8	211.6	782.3	1861.0

Table S2. Linear regression analysis for ln (C_{IL-10})

Variable	β	*CI95%*		*SE*	$\beta_{stand.}$	*t*	*p*
**(constant)**	3.368	1.742	4.993	0.813	-0.104	4.141	<0.001
**Alzheimer**	1.962	0.269	3.656	0.848	0.570	2.315	0.024
$\sqrt{API}$	0.068	-1.372	1.509	0.721	-0.057	0.095	0.925
**age**	0.039	0.015	0.062	0.012	0.314	3.276	0.002
$\sqrt{API}$**×Alzheimer**	-2.163	-4.296	-0.031	1.067	-0.628	-2.028	0.047

$F(3;64) = 4.457$; $p = 0.003$; adjusted $R^2 = 0.171$. The normality of the random component was assessed using the Shapiro-Wilk test ($W = 0.967$; $p = 0.067$). homoscedasticity of the random component was examined using the Harrison-McCabe test ($b = 0.395$; $p = 0.106$). and the linearity of the model was determined using the Wald-Wolfowitz runs test ($Z = -0.816$; $p = 0.415$). No influential observations were detected (∀i Cook's distance < $F(3;68) = 0.32$ for α

= 0.1). $\sqrt{API}$ – normalized (transformation: square root) interproximal hygiene index; Alzheimer – nominal variable classifying individuals into cognitively healthy [0] or diseased [1] groups; β – unstandardized linear regression coefficient; SE – standard error; βstand. – standardized linear regression coefficient; t – t-test statistic; F – ANOVA result; CI95% – 95% confidence interval. The dependent variable: natural logarithm of interleukin 10 concentration. Values are rounded to three decimal places.

Table S3. Linear regression analysis for ln (C_{IL-6})

Variable	*β*	*CI95%*		*SE*	*$β_{stand.}$*	*t*	*p*
**(constant)**	8.485	7.248	9.722	0.618	0.322	13.724	<0.001
**Alzheimer**	1.812	0.495	3.130	0.659	0.189	2.752	0.008
**$\sqrt{API}$**	0.363	-0.823	1.549	0.593	0.324	0.612	0.543
**age**	0.016	-0.001	0.033	0.008	0.165	1.840	0.071
**$\sqrt{API}$×Alzheimer**	-2.324	-4.000	-0.648	0.838	-1.106	-2.774	0.007

F(3;64) = 3.57; p = 0.011; adjusted R^2 = 0.14. The normality of the random component was assessed using the Shapiro-Wilk test (W = 0.95; p = 0.016). homoscedasticity of the random component was examined using the Harrison-McCabe test (b = 0.56; p = 0.777). and the linearity of the model was determined using the Wald-Wolfowitz runs test (Z = -3.03; p = 0.002). No influential observations were detected (∀i Cook's distance < F(3;68) = 0.32 for α = 0.1). $\sqrt{API}$ – normalized (transformation: square root) interproximal hygiene index; Alzheimer – nominal variable classifying individuals into cognitively healthy [0] or diseased [1] groups; β – unstandardized linear regression coefficient; SE – standard error; βstand. – standardized linear regression coefficient; t – t-test statistic; F – ANOVA result; CI95% – 95% confidence interval. The dependent variable: natural logarithm of interleukin 1 concentration. Values are rounded to three decimal places.
